# Early prediction of level-of-care requirements in patients with COVID-19

**DOI:** 10.7554/eLife.60519

**Published:** 2020-10-12

**Authors:** Boran Hao, Shahabeddin Sotudian, Taiyao Wang, Tingting Xu, Yang Hu, Apostolos Gaitanidis, Kerry Breen, George C Velmahos, Ioannis Ch Paschalidis

**Affiliations:** 1 Center for Information and Systems Engineering, Boston UniversityBostonUnited States; 2 Division of Trauma, Emergency Services, and Surgical Critical Care Massachusetts General Hospital, Harvard Medical SchoolBostonUnited States; Attikon University HospitalGreece; Radboud University Medical CentreNetherlands

**Keywords:** COVID-19, SARS-CoV-2, risk prediction, artificial intelligence, machine learning, critical care, Human

## Abstract

This study examined records of 2566 consecutive COVID-19 patients at five Massachusetts hospitals and sought to predict level-of-care requirements based on clinical and laboratory data. Several classification methods were applied and compared against standard pneumonia severity scores. The need for hospitalization, ICU care, and mechanical ventilation were predicted with a validation accuracy of 88%, 87%, and 86%, respectively. Pneumonia severity scores achieve respective accuracies of 73% and 74% for ICU care and ventilation. When predictions are limited to patients with more complex disease, the accuracy of the ICU and ventilation prediction models achieved accuracy of 83% and 82%, respectively. Vital signs, age, BMI, dyspnea, and comorbidities were the most important predictors of hospitalization. Opacities on chest imaging, age, admission vital signs and symptoms, male gender, admission laboratory results, and diabetes were the most important risk factors for ICU admission and mechanical ventilation. The factors identified collectively form a signature of the novel COVID-19 disease.

## eLife digest

The new coronavirus (now named SARS-CoV-2) causing the disease pandemic in 2019 (COVID-19), has so far infected over 35 million people worldwide and killed more than 1 million. Most people with COVID-19 have no symptoms or only mild symptoms. But some become seriously ill and need hospitalization. The sickest are admitted to an Intensive Care Unit (ICU) and may need mechanical ventilation to help them breath. Being able to predict which patients with COVID-19 will become severely ill could help hospitals around the world manage the huge influx of patients caused by the pandemic and save lives.

Now, Hao, Sotudian, Wang, Xu et al. show that computer models using artificial intelligence technology can help predict which COVID-19 patients will be hospitalized, admitted to the ICU, or need mechanical ventilation. Using data of 2,566 COVID-19 patients from five Massachusetts hospitals, Hao et al. created three separate models that can predict hospitalization, ICU admission, and the need for mechanical ventilation with more than 86% accuracy, based on patient characteristics, clinical symptoms, laboratory results and chest x-rays.

Hao et al. found that the patients’ vital signs, age, obesity, difficulty breathing, and underlying diseases like diabetes, were the strongest predictors of the need for hospitalization. Being male, having diabetes, cloudy chest x-rays, and certain laboratory results were the most important risk factors for intensive care treatment and mechanical ventilation. Laboratory results suggesting tissue damage, severe inflammation or oxygen deprivation in the body's tissues were important warning signs of severe disease.

The results provide a more detailed picture of the patients who are likely to suffer from severe forms of COVID-19. Using the predictive models may help physicians identify patients who appear okay but need closer monitoring and more aggressive treatment. The models may also help policy makers decide who needs workplace accommodations such as being allowed to work from home, which individuals may benefit from more frequent testing, and who should be prioritized for vaccination when a vaccine becomes available.

## Introduction

As a result of the SARS-CoV-2 pandemic, many hospitals across the world have resorted to drastic measures: canceling elective procedures, switching to remote consultations, designating most beds to COVID-19, expanding Intensive Care Unit (ICU) capacity, and re-purposing doctors and nurses to support COVID-19 care. In the U.S., the CDC estimates more than 310,000 COVID-19 hospitalizations from March 1 to June 13, 2020 (CDC, 2020).

Much of the modeling work related to the pandemic has focused on spread dynamics (Kucharski et al., 2020). Others have described patients who were hospitalized (Richardson et al., 2020) (n = 5700) and (Buckner et al., 2020) (n = 105), became critically ill (Gong et al., 2020) (n = 372), or succumbed to the disease (n = 1625 (Onder et al., 2020), n = 270 [Wu et al., 2020]). In data from the New York City, 14.2% required ICU treatment and 12.2% mechanical ventilation (Richardson et al., 2020). With such rates, the logistical and ethical implications of bed allocation and potential rationing of care delivery are immense (White and Lo, 2020). To date, while state- or country-level prognostication has developed to examine resource allocation at a mass scale, there is inadequate evidence based on a large cohort on accurate prediction of the disease progress at the individual patient level. A string of recent studies developed models to predict severe disease or mortality based on clinical and laboratory findings, for example (Yan et al., 2020) (n = 485), (Gong et al., 2020) (n = 372), (Bhargava et al., 2020) (n = 197), (Ji et al., 2020) (n = 208), and (Wang et al., 2020) (n = 296). In these studies, several variables such as Lactate Dehydrogenase (LDH) (Gong et al., 2020; Ji et al., 2020; Yan et al., 2020) and C-reactive protein (CRP) have been identified as important predictors. All of these studies considered relatively small cohorts and, with the exception of Bhargava et al., 2020, considered patients in China. Although it is believed that the virus remains the same around the globe, the physiologic response to the virus and the eventual course of disease depend on multiple other factors, many of them regional (e.g. population characteristics, hospital practices, prevalence of pre-existing conditions) and not applicable universally. Triage of adult patients with COVID-19 remains challenging with most evidence coming from expert recommendations; evidence-based methods based on larger U.S.-based cohorts have not been reported (Sprung et al., 2020).

Leveraging data from five hospitals of the largest health care system in Massachusetts, we seek to develop personalized, interpretable predictive models of (i) hospitalization, (ii) ICU treatment, and (iii) mechanical ventilation, among SARS-CoV-2 positive patients. To develop these models, we developed a pipeline leveraging state-of-the-art Natural Language Processing (NLP) tools to extract information from the clinical reports for each patient, employing statistical feature selection methods to retain the most predictive features for each model, and adapting a host of advance machine learning-based classification methods to develop parsimonious (hence, easier to use and interpret) predictive models. We found that the more interpretable models can, for the most part, deliver similar predictive performance compared to more complex, ‘black-box’ models involving ensembles of many decision trees. Our results support our initial hypothesis that important clinical outcomes can be predicted with a high degree of accuracy upon the patient’s *first* presentation to the hospital using a relatively small number of features, which collectively compose a ‘signature’ of the novel COVID-19 disease.

## Results

We extracted data for all patients (n = 2566) who had a positive RT-PCR SARS-CoV-2 test between March 4 and April 13, 2020 at five Massachusetts hospitals, included in the same health care system (Massachusetts General Hospital (MGH), Brigham and Women’s Hospital (BWH), Faulkner Hospital (FH), Newton-Wellesley Hospital (NWH), and North Shore Medical Center (NSM)). The study was approved by the pertinent Institutional Review Boards.

Demographics, pre-hospital medications, and comorbidities were extracted for each patient based on the electronic medical record. Patient symptoms, vital signs, radiologic findings, and laboratory results were recorded at their first hospital presentation (either clinic or emergency department) before testing positive for SARS-CoV-2. A total of 164 features were extracted for each patient. ICU admission and mechanical ventilation were determined for each patient. Complete blood count values were considered as absolute counts. Representative statistics comparing hospitalized, ICU admitted, and mechanically ventilated patients are provided in Table A1 (Appendix). Table A2 (Appendix) reports how patients were distributed among the five hospitals.

Among the 2566 patients with a positive test, 930 (36.2%) were hospitalized. Among the hospitalized, 273 (29.4% of the hospitalized) required ICU care of which 217 (79.5%) required mechanical ventilation. The mean age over all patients was 51.9 years (SD: 18.9 years) and 45.6% were male.

### Hospitalization

The mean age of hospitalized patients was 62.3 years (SD: 18 years) and 55.3% were male. We employed linear and non-linear classification methods for predicting hospitalizations. Non-linear methods included random forests (RF) (Breiman, 2001) and XGBoost (Chen and Guestrin, 2016). Linear methods included support vector machines (SVM) (Cortes and Vapnik, 1995) and Logistic Regression (LR); each linear method used either ℓ_1_- or ℓ_2_-norm regularization and we report the best-performing flavor of each model.

Results are reported in Table 1. We report the Area Under the Curve (AUC) of the Receiver Operating Characteristic (ROC) and the Weighted-F1 score, both computed out-of-sample (in a test set not used for training the model). As we detail under Methods, we used two validation strategies. The ‘Random’ strategy randomly split the patients into a training and a test set and was repeated five times; from these five splits we report the average and the standard deviation of the test performance. The ‘BWH’ strategy trained the models on MGH, FH, NWH, and NSM patients, and evaluated performance on BWH patients.

**Table 1.** Hospitalization prediction model (test performance). The values inside the parentheses refer to the standard deviation of the corresponding metric. Random refers to test set results from the five random training/test splits. BWH refers to training on four other hospitals and testing on data from BWH. SVM-L1 and LR-L1 refer to the ℓ_1_-norm regularized SVM and LR models. For the parsimonious model, we list the LR coefficients of each variable (*Coef*), the correlation of the variable with the outcome (*Y-corr*), the mean of the variable (*Y1-mean*) in the positive class (hospitalized for this table), and the mean of the variable (*Y0-mean*) in the negative class (non-hospitalized). *Binary Coef* denotes the coefficient of the variables in the binarized model. We report the corresponding odds ratio (OR) and the 95% confidence intervals (CI). Thresholds used for the binarized model are provided in Appendix 1—table 5.

Algorithm		AUC				F1-weighted			
		Random		BWH		Random		BWH	
		Models using all 106 features							
LR-L2		87.0% (1.7%)		85.9%		81.6% (1.3%)		84.2%	
SVM-L1		87.0% (1.6%)		85.8%		81.5% (1.5%)		83.9%	
XGBoost		87.8% (1.9%)		87.7%		80.9% (1.8%)		83.3%	
RF		88.2% (1.6%)		88.1%		81.2% (1.1%)		83.2%	
		Models using 74 statistically selected features							
LR-L2		87.1% (1.7%)		86.0%		82.0% (1.3%)		83.9%	
SVM-L1		87.1% (1.7%)		85.8%		82.0% (1.4%)		84.0%	
XGBoost		87.9% (1.9%)		87.6%		81.2% (1.9%)		84.2%	
RF		88.0% (1.7%)		88.1%		80.8% (1.7%)		83.9%	
		Parsimonious Model using 11 features							
LR-L2		83.4% (1.7%)		83.7%		78.7% (0.9%)		81.0%	
SVM-L1		83.4% (1.7%)		83.8%		78.1% (1.1%)		79.9%	
Variables for the Parsimonious Model									
Variable	Coef	Y1 mean	Y0 mean	p-value	Y-corr	Coef binary	OR	OR 95% CI	
SpO2 (%)	−11.90	95.44	97.11	<0.001	−0.29	1.74	5.67	3.97	8.12
Temperature	10.36	37.21	37.06	<0.001	0.08	0.86	2.36	1.76	3.18
Respiratory Rate	7.20	22.82	20.83	<0.001	0.18	−0.13	0.88	0.69	1.13
Age	5.14	62.31	46.02	<0.001	0.41	0.88	2.4	1.86	3.11
Pulse	4.60	90.09	90.4	<0.001	−0.01	0.7	2.01	1.49	2.71
Diastolic BP	−3.56	73.07	77.21	<0.001	−0.23	1.51	4.51	2.88	7.06
Adrenal Insufficiency	3.09	0.013	0.001	<0.001	0.08	2.58	13.14	1.57	110.37
BMI	2.30	31.34	31.64	<0.001	−0.04	−0.09	0.91	0.71	1.17
Transplantation	1.90	0.023	0.002	<0.001	0.1	1.43	4.19	1.04	16.87
Dyspnea	1.85	0.17	0.02	<0.001	0.26	2	7.41	4.85	11.32
CKD	1.55	0.14	0.02	<0.001	0.25	0.81	2.25	1.35	3.74
Intercept	−2.51								

SpO2: oxygen saturation; BP: Blood pressure; BMI: Body Mass Index; CKD: Chronic Kidney Disease.

The hospitalization models used symptoms, pre-existing medications, comorbidities, and patient demographics. Laboratory results and radiologic findings were not considered since these were not available for most non-hospitalized patients. Full models used all (106) variables retained after several pre-processing steps described in Materials and methods. Applying the statistical variable selection procedure described in the Appendix (specifically, eliminating variables with a p-value exceeding 0.05), yields a model with 74 variables. To provide a more parsimonious, highly interpretable, and easier to implement model, we used recursive feature elimination (see Appendix) to select a model with only 11 variables. The best model using the random validation approach has an AUC of 88% while the best parsimonious (linear) model has an AUC of 83%, being though easier to interpret and implement. Validation on the BWH patients yields an AUC of 84% for the parsimonious model.

Table 1 also reports the 11 variables in the parsimonious LR model, including their LR coefficients, and a binarized version of this model as described in Materials and methods. The most important variables associated with hospitalization were: oxygen saturation, temperature, respiratory rate, age, pulse, blood pressure, a comorbidity of adrenal insufficiency, BMI, prior transplantation, dyspnea, and kidney disease.

Additionally, we assessed the role of pre-existing ACE inhibitor (ACEI) and angiotensin receptor blocker (ARB) medications by adding these variables into the parsimonious binarized model, while controlling for additional relevant variables (hypertension, diabetes, and arrhythmia comorbidities and other hypertension medications). We found that while ARBs are not a factor, ACEIs reduce the odds of hospitalization by 3/4, on average, controlling for other important factors, such as age, hypertension, and related comorbidities associated with the use of these medications.

### ICU admission

The mean age of ICU admitted patients was 63.3 years (SD: 15.1 years) and 63% were male. The ICU and ventilation prediction models used the features considered for the hospitalization, as well as laboratory results and radiologic findings. For these models, we excluded patients who required immediate ICU admission or ventilation (defined as within 4 hr from initial presentation). This was implemented in order to focus on patients where triaging is challenging and risk prediction would be beneficial. There were 2513 and 2525 patients remaining for the ICU and the mechanical ventilation prediction models, respectively.

For the model including 2513 patients (Table 2), we first developed a model using all 130 variables retained after pre-processing, then employed statistical variable selection to retain 56 of the variables, and then applied recursive feature elimination with LR to select a parsimonious model which uses only 10 variables. The following variables were included: opacity observed in a chest scan, respiratory rate, age, fever, male gender, albumin, anion gap, oxygen saturation, LDH, and calcium. In addition, we generated a binarized version of the parsimonious model. The parsimonious model for all 2513 patients has an AUC of 86%, almost as high as the model with all 130 features.

**Table 2.** ICU prediction model (test performance). Abbreviations are as in Table 1. Thresholds for the binarized model, PSI and CURB-65 scores are in the Appendix.

ICU prediction results with 2513 patients									
Algorithm		AUC				F1-weighted			
		Random		BWH		Random		BWH	
		Models using all 130 features							
XGBoost		86.0% (2.8%)		83.1%		90.0% (1.7%)		91.7%	
SVM-L1		85.9% (2.5%)		80.2%		89.9% (1.0%)		89.2%	
LR-L1		84.6% (2.8%)		76.8%		89.7% (1.0%)		89.9%	
RF		86.9% (2.4%)		83.7%		90.4% (1.1%)		91.1%	
		Models using 56 statistically selected features							
XGBoost		86.8% (3.1%)		82.8%		90.4% (1.4%)		91.3%	
SVM-L1		86.2% (2.6%)		82.6%		90.6% (1.2%)		90.8%	
LR-L1		85.8% (2.9%)		81.8%		90.2% (1.3%)		91.3%	
RF		86.7% (2.0%)		83.2%		90.5% (1.7%)		91.5%	
		Parsimonious Model using 10 features							
LR-L1		85.8% (2.6%)		83.9%		90.0% (1.4%)		89.1%	
LR-L1 (binarized model)		84.2% (2.2%)		82.5%		89.8% (1.1%)		88.1%	
		Model using PSI or CURB-65 score							
PSI score		72.9% (4.9%)		78.8%		86.8% (0.7%)		88.2%	
CURB-65 score		67.0% (5.0%)		75.4%		87.0% (0.5%)		88.1%	
Variables for the parsimonious model									
Variable	Coef	Y1 mean	Y0 mean	p-value	Y-corr	Coef binary	OR	OR 97.5% CI	
Radiology Opacities	0.54	0.76	0.27	<0.001	0.30	1.41	4.08	2.83	5.89
Respiratory Rate	0.46	24.61	21.37	<0.001	0.16	0.50	1.66	1.14	2.41
Age	0.45	62.61	50.58	<0.001	0.18	0.56	1.76	1.27	2.43
Fever	0.40	0.64	0.33	<0.001	0.18	0.61	1.83	1.32	2.55
Male	0.35	0.64	0.44	<0.001	0.12	0.50	1.65	1.21	2.26
Albumin	−0.34	3.68	3.84	<0.001	−0.16	0.58	1.78	1.10	2.90
Anion Gap	0.33	16.40	15.35	<0.001	0.13	−0.05	0.95	0.46	1.98
SpO2 (%)	−0.22	94.72	96.72	<0.001	−0.24	0.83	2.29	1.63	3.21
LDH	0.22	400.40	327.48	<0.001	0.15	0.96	2.62	1.74	3.94
Calcium	−0.21	8.84	9.01	<0.001	−0.10	0.55	1.73	1.21	2.48
Intercept	−0.93								

SpO2: oxygen saturation; LDH: Lactate dehydrogenase.

For comparison purposes against well-established scoring systems, we implemented two commonly used pneumonia severity scores, CURB-65 (Lim et al., 2003) and the Pneumonia Severity Index (PSI) (Fine et al., 1997). Predictions based on the PSI and CURB-65 scores, have AUCs of 73% and 67%, respectively.

We also developed a model for a more restrictive set of patients. Specifically, the number of missing lab values for some patients is substantial. Given the importance of LDH and CRP, as revealed by our models, the more restricted patient set contains 669 patients with non-missing LDH and CRP values. After removing patients who required intubation or ICU admission within 4 hr of hospital presentation, we included 628 patients and 635 patients for the restricted ICU admission and ventilation models, respectively.

The best restricted model for the 628 patients (Table 3) is the nonlinear XGBoost model using 29 statistically selected features with an AUC of 83%, with a linear parsimonious LR model close behind (AUC 80%). An RF model using all variables yields an AUC of 77% when tested on BWH data. PSI- and CURB-65 models have AUCs below 59%.

**Table 3.** Restricted ICU prediction model (test performance). Abbreviations are as in Table 1. Thresholds for the binarized model, PSI and CURB-65 scores are in the Appendix.

ICU prediction results with 628 patients									
Algorithm		AUC				F1-weighted			
		Random		BWH		Random		BWH	
		Models using all 130 features							
XGBoost		82.5% (1.9%)		67.3%		81.4% (0.7%)		72.6%	
SVM-L1		77.8% (3.8%)		72.8%		79.7% (1.2%)		73.6%	
LR-L1		75.9% (3.6%)		69.7%		79.2% (2.5%)		73.7%	
RF		80.9% (2.7%)		76.9%		78.8% (1.9%)		73.6%	
		Models using 29 statistically selected features							
XGBoost		82.7% (2.7%)		76.2%		80.6% (2.1%)		72.6%	
SVM-L1		77.9% (3.7%)		73.1%		78.5% (1.4%)		73.6%	
LR-L1		78.4% (4.1%)		71.5%		79.5% (2.6%)		74.4%	
RF		82.1% (2.8%)		74.1%		79.0% (2.4%)		75.4%	
		Parsimonious Model using 8 features							
LR-L1		80.1% (2.9%)		74.2%		80.9% (2.1%)		77.2%	
LR-L1 (binarized model)		72.5% (5.4%)		69.9%		73.4% (2.8%)		69.7%	
		Model using PSI or CURB-65 score							
PSI score		58.8% (7.4%)		68.3%		66.7% (2.2%)		65.3%	
CURB-65 score		56.8% (4.5%)		76.9%		66.2% (1.5%)		63.8%	
Variables for the parsimonious model									
Variable	Coef	Y1 mean	Y0 mean	p-value	Y-corr	Coef binary	OR	OR 97.5% CI	
LDH	0.53	519.88	304.40	<0.001	0.15	1.59	4.88	2.65	8.99
CRP (mg/L)	0.47	127.17	67.43	<0.001	0.35	0.76	2.13	0.70	6.47
Calcium	−0.35	8.83	9.01	<0.001	−0.13	0.71	2.03	1.25	3.31
IDDM	0.30	0.25	0.12	0.003	0.15	1.00	2.73	1.62	4.60
SpO2 (%)	−0.29	94.13	95.59	0.003	−0.22	0.34	1.41	0.92	2.16
Radiology Opacities	0.25	0.88	0.71	<0.001	0.16	0.62	1.86	1.05	3.29
Anion Gap	0.20	16.66	15.28	<0.001	0.20	0.34	1.40	0.48	4.12
Sodium	−0.16	136.13	137.53	<0.001	−0.14	0.47	1.60	1.05	2.43
Intercept	−0.34								

LDH: Lactate dehydrogenase; CRP: C-reactive protein; IDDM: Insulin-dependent diabetes mellitus; SpO2: oxygen saturation.

### Mechanical ventilation

The mean age of patients requiring mechanical ventilation was 63.3 years (SD: 14.7 years) and 63.6% were male. Again, we excluded patients who were intubated within 4 hr of their hospital admission.

For the model including 2525 patients (Table 4), we used statistical feature selection to select 55 variables, and recursive feature elimination with LR to select a parsimonious model with only eight variables. The following variables were included: lung opacities, albumin, fever, respiratory rate, glucose, male gender, LDH, and anion gap. In addition, we generated a binarized version of the parsimonious model. The best model for all 2525 patients was a nonlinear RF model using the 55 statistically selected variables and yielding an AUC of 86%. The best linear model was the parsimonious LR model with an AUC of 85%. PSI- and CURB-65 models yield AUCs of 74% and 67%, respectively.

**Table 4.** Ventilation prediction model (test performance). Abbreviations are as in Table 1. Thresholds for the binarized model, PSI and CURB-65 scores are in the Appendix.

Ventilation prediction results with 2525 patients									
Algorithm		AUC				F1-weighted			
		Random		BWH		Random		BWH	
		Models using all 130 features							
XGBoost		85.8% (4.0%)		83.8%		91.0% (0.4%)		91.6%	
SVM-L1		82.6% (4.9%)		83.8%		90.9% (0.8%)		91.6%	
LR-L1		80.7% (5.4%)		81.7%		90.4% (1.2%)		91.4%	
RF		85.7% (3.9%)		83.7%		91.2% (0.9%)		91.8%	
		Models using 55 statistically selected features							
XGBoost		85.7% (3.3%)		86.3%		91.1% (0.6%)		91.6%	
SVM-L1		83.9% (3.7%)		84.8%		90.9% (1.1%)		91.7%	
LR-L1		83.3% (4.0%)		83.9%		90.8% (1.3%)		91.4%	
RF		86.4% (3.4%)		86.7%		91.4% (1.1%)		91.3%	
		Parsimonious Model using 8 features							
LR-L1		85.2% (2.3%)		87.0%		90.3% (0.3%)		90.7%	
LR-L1 (binarized model)		81.3% (3.1%)		82.6%		90.0% (0.6%)		90.2%	
		Model using PSI or CURB-65 score							
PSI score		73.6% (4.1%)		80.7%		89.4% (0.4%)		90.3%	
CURB-65 score		66.8% (3.1%)		75.9%		89.7% (0.1%)		90.0%	
Variables for the Parsimonious Model									
Variable	Coef	Y1 mean	Y0 mean	p-value	Y-corr	Coef binary	OR	OR 97.5% CI	
Radiology opacities	0.86	0.77	0.28	<0.001	0.27	1.58	4.86	3.25	7.25
Albumin	−0.45	3.65	3.83	<0.001	−0.16	1.07	2.91	1.80	4.72
Fever	0.43	0.66	0.33	<0.001	0.17	0.72	2.05	1.42	2.95
Respiratory rate	0.42	24.70	21.44	<0.001	0.15	0.50	1.64	1.09	2.47
Glucose	0.38	170.17	138.32	<0.001	0.15	0.97	2.63	1.71	4.06
Male	0.34	0.64	0.44	<0.001	0.10	0.43	1.54	1.09	2.18
LDH	0.33	408.56	328.78	<0.001	0.14	0.91	2.48	1.58	3.89
Anion gap	0.31	16.50	15.37	<0.001	0.13	0.27	1.31	0.53	3.25
Intercept	−1.06								

LDH: Lactate dehydrogenase.

The best model for the restricted case of 635 patients (Table 5) was the linear parsimonious LR model (with just five variables) achieving an AUC of 82%. PSI- and CURB-65 models do not exceed AUC of 58%.

**Table 5.** Restricted ventilation prediction model (test performance). Abbreviations are as in Table 1.Thresholds for the binarized, PSI and CURB-65 scores are in the Appendix.

Ventilation prediction results with 635 patients									
Algorithm		AUC				F1-weighted			
		Random		BWH		Random		BWH	
		Models using all 130 features							
XGBoost		80.6% (1.9%)		74.7%		79.4% (2.6%)		75.7%	
SVM-L1		79.4% (5.2%)		71.3%		80.8% (2.0%)		75.7%	
LR-L1		76.9% (3.9%)		68.2%		78.6% (3.2%)		73.4%	
RF		81.0% (3.1%)		75.8%		79.8% (4.2%)		72.7%	
		Models using 29 statistically selected features							
XGBoost		81.6% (3.2%)		76.9%		79.0% (2.9%)		71.7%	
SVM-L1		79.1% (4.6%)		69.4%		80.6% (2.5%)		75.7%	
LR-L1		80.9% (3.6%)		70.9%		80.4% (2.2%)		75.7%	
RF		81.3% (2.6%)		75.4%		79.2% (1.7%)		69.6%	
		Parsimonious Model using 5 features							
LR-L1		82.4% (3.7%)		75.2%		81.8% (1.7%)		71.7%	
LR-L1 (binarized model)		71.4% (6.2%)		65.5%		76.6% (3.5%)		68.3%	
		Model using PSI or CURB-65 score							
PSI score		57.6% (4.5%)		67.4%		73.2% (1.3%)		71.2%	
CURB-65 score		56.9% (7.1%)		74.0%		72.4% (0.2%)		68.3%	
Variables for the parsimonious model									
Variable	Coef	Y1 mean	Y0 mean	p-value	Y-corr	Coef binary	OR	OR 97.5% CI	
CRP (mg/L)	0.60	134.52	69.62	<0.001	0.35	0.42	1.53	0.51	4.59
LDH	0.55	550.41	311.01	<0.001	0.16	1.87	6.47	3.19	13.10
Calcium	−0.39	8.82	9.00	<0.001	−0.13	0.58	1.79	1.07	2.98
IDDM	0.36	0.26	0.12	0.002	0.15	1.18	3.26	1.90	5.58
Anion Gap	0.29	16.81	15.32	<0.001	0.19	18.66	1.27E+08	0.00	inf
Intercept	−0.39								

CRP: C-reactive protein; LDH: Lactate dehydrogenase; IDDM: Insulin-dependent diabetes mellitus.

### Time period between ICU/ventilation model prediction and corresponding outcomes

Table 6 reports the mean and the median time interval (in hours) between hospital admission time and ICU/ventilation outcomes. Specifically, we report statistics for ICU admission or intubation outcomes from the correct ICU/intubation predictions made by our models trained on four hospitals (MGH, NWH, NSM, FH) and applied to BWH patients (both the models making predictions for all patients and the restricted models). As we have noted earlier, our models use the lab results closest to admission (either on admission date or the following day). We also report the time interval between the last lab result used by the model and the corresponding ICU/intubation outcome.

**Table 6.** Mean and median hours between reference date/lab results to outcomes in full/restricted ICU and ventilation model prediction.

	From reference date (mean)	From reference date (median)	From lab results (mean)	From lab results (median)
Restricted ICU	38.13	28.08	22.55	9.90
Restricted intubation	35.36	26.40	22.37	10.39
Full ICU	22.86	17.28	15.86	12.99
Full intubation	25.62	22.20	10.23	8.97

## Discussion

We developed three models to predict need for hospitalization, ICU admission, and mechanical ventilation in patients with COVID-19. The prediction models are not meant to replace clinicians’ judgment for determining level of care. Instead, they are designed to assist clinicians in identifying patients at risk of future decompensation. Patient vital signs were the most important predictors of hospitalization. This is expected as vital signs reflect underlying disease severity, the need for cardiorespiratory resuscitation, and the risk of future decompensation without adequate medical support. Older age and BMI were also important predictors for hospitalization. Age has been recognized as an important factor associated with severe COVID-19 in previous series (Grasselli et al., 2020; Guan et al., 2020; Richardson et al., 2020). However, it is not known whether age itself or the presence of comorbidities place patients at risk for severe disease. Our results demonstrate that age is a stronger predictor of severe COVID-19 than a host of underlying comorbidities.

In terms of patient comorbidities, adrenal insufficiency, prior transplantation, and chronic kidney disease were strongly associated with need for hospitalization. Diabetes mellitus was associated with a need for ICU admission and mechanical ventilation, which might be due to its detrimental effects on immune function.

For the ICU and ventilation prediction models screening all at-risk (COVID-19-positive patients), opacities observed in a chest scan, age, and male gender emerge as important variables. Males have been found to have worse in-hospital outcomes in other studies as well (Palaiodimos et al., 2020).

We also identified several routine laboratory values that are predictive of ICU admission and mechanical ventilation. Elevated serum LDH, CRP, anion gap, and glucose, as well as decreased serum calcium, sodium, and albumin were strong predictors of ICU admission and mechanical ventilation. LDH is an indicator of tissue damage and has been found to be a marker of severity in P. jirovecii pneumonia (Zaman and White, 1988). Along with CRP, it was among the two most important predictors of ICU admission and ventilation in the parsimonious model among patients who had LDH and CRP measurements on admission. This finding is consistent with previous reports identifying LDH as an important prognostic factor (Gong et al., 2020; Ji et al., 2020; Mo et al., 2020; Yan et al., 2020). In addition, lower serum calcium is associated with cell lysis and tissue destruction, as it is often seen as part of the tumor lysis syndrome. Elevated serum anion gap is a marker of metabolic acidosis and ischemia, suggesting that tissue hypoxia and hypoperfusion may be components of severe disease.

For all three prognostic models, we developed predicting hospitalizations, ICU care, and mechanical ventilation, AUC ranges within 86–88%, which indicates strong predictive power. Interestingly, we can achieve AUC within 85–86% for ICU and ventilation prediction with a parsimonious linear model utilizing no more than 10 variables. In all cases, we can also develop a parsimonious model with binarized variables using medically suggested normal and abnormal variable thresholds. These binarized models have similar performance with their continuous counterparts. The ICU and ventilation models using all patients are very accurate, but, arguably, make a number of ‘easier’ decisions since more than 60% of the patients are never hospitalized. Many of these patients are younger, healthy, and likely present with mild-to-moderate symptoms. To test the robustness of the models to patients with potentially more ‘complex’ disease, we developed ICU and ventilation models on a restricted set of patients. This is the subset of patients who are hospitalized and most of the crucial labs are available for them (specifically CRP and LDH which emerged as important from our models). The best AUC for these models drops, but not below 82%, which indicates robustness of the model even when dealing with arguably harder to assess cases. LDH, CRP, calcium, lung opacity, anion gap, SpO2, sodium, and a comorbidity of insulin-controlled diabetes appear as the most significant for these patients. Interestingly, the corresponding binarized models have about 10% lower AUC; apparently, for the more severely ill, clinical variables deviate substantially from normal and knowing the exact values is crucial.

The models have been validated with two different approaches, using random splits of the data into training and testing, as well as training in some hospitals and testing at a different hospital. Performance metrics are relatively consistent with these two approaches. We also compared the models against standard pneumonia severity scores, PSI and CURB-65, establishing that our models are significantly stronger, which highlights the different clinical profile of COVID-19.

We also examined how much in advance of the ICU or ventilation outcomes our models are able to make a prediction. Of course, this is not entirely in our control; it depends on what state the patients get admitted and how soon their condition deteriorates to require ICU admission and/or ventilation. Table 6 reports the corresponding statistics. For example, the restricted ICU and ventilation models are making a correct prediction upon admission (using the lab results closest to that time) for outcomes that on average occur 38 and 35 hr later, respectively.

To further test the accuracy of the restricted ICU and ventilation models well in advance of the corresponding event, we considered an extended BWH test set (adding 11 more patients) and computed the accuracy of the models when the test set was restricted to patients whose outcome (ICU admission or ventilation) was more than x hours after the admission lab results based on which the prediction was made, with x being 6 hr, or 12 hr, or 18 hr, or 24 hr, or even 48 hr. The ICU model reaches an AUC of 87% and a weighted F1-score of 86% at x = 18 hr. The ventilation model reaches an AUC of 64% and an F1-score of 72% at x = 48 hr. These results demonstrate that the predictive models can indeed make predictions well into the future, when physicians would be less certain about the course of the disease and when there is potentially enough time to intervene and improve outcomes.

A manual review of the predictions by the models indicates that they performed well at predicting future ICU admissions for patients who presented with mild disease several days before ICU admission was necessary. Such patients were hemodynamically stable and had minimal oxygen requirements on the floor, before clinical deterioration necessitated ICU admission. We identified several such patients. A typical case is that of a 51-year-old male with a history of hypertension, obesity, and insulin-dependent type 2 diabetes mellitus, who presented with a 3-day history of dyspnea, cough and myalgias. In the emergency department, he was hemodynamically stable, saturating at 96–97% on 2 L of nasal cannula. The patient was admitted to the floor and did well for 3 days, saturating at 93–96% on room air. On the fourth day of hospitalization, he had increasing oxygen requirements and the decision was made to transfer him to the ICU. He was intubated and ventilated for 30 days. Our prediction models accurately predicted at the time of his presentation that he would eventually require ICU admission and mechanical ventilation. This prediction was based on such variables as an elevated LDH (241 U/L) and the presence of insulin-dependent diabetes mellitus. Another such case is that of a 59-year-old male without a significant prior medical history who presented with 2 days of dyspnea, nausea, and diarrhea. At the emergency department, he was tachycardic at 110 beats per minute and saturating at 96% on room air, and the patient was admitted. For 2 days, the patient was hemodynamically stable, saturating at 94–97% on room air. On the third day of hospitalization, he had increasing oxygen requirements, eventually requiring transfer to the ICU. He was intubated and ventilated for the next 14 days. Our prediction model predicted the patient’s decompensation at his presentation, due to elevations in LDH (348 U/L) and CRP (102.3 mg/L).

We also considered the role of ACEIs and ARBs and their potential association with the outcomes. It has been speculated that ACEIs may worsen COVID-19 outcomes because they upregulate the expression of ACE2, which the virus targets for cell entry. No such evidence has been reported in earlier studies (Kuster et al., 2020; Patel and Verma, 2020). In fact, a smaller study (Zhang et al., 2020) (n = 1128 vs. 2566 in our case) reported a beneficial effect and (Rossi et al., 2020) warn of potential harmful effects of discontinuing ACEIs or ARBs due to COVID-19. Our hospitalization model suggests that ACEIs do not increase hospitalization risk and may slightly reduce it (OR 95% CI is (0.52,1.04) with a mean of 0.73). In the ICU and ventilation models, the role of these two medications is statistically weaker to observe any meaningful association.

The models we derived can be used for a variety of purposes: (i) guiding patient triage to appropriate inpatient units, (ii) guiding staffing and resource planning logistics, and (iii) understanding patient risk profiles to inform future policy decisions, such as targeted risk-based stay-at-home restrictions, testing, and vaccination prioritization guidelines once a vaccine becomes available.

Calculators implementing the parsimonious models corresponding to each of the Tables 1, 2, 3, 4, 5 have been made available online (Hao et al., 2020).

## Materials and methods

### Data extraction

Natural Language Processing (NLP) was used to extract patient comorbidities (see Appendix for details), pre-existing medications, admission vital signs, hospitalization course, ICU admission, and mechanical intubation.

### Pre-processing

The categorical features were converted to numerical by ‘one-hot’ encoding. Each categorical feature, such as gender and race, was encoded as an indicator variable for each category. Features were standardized by subtracting the mean and dividing by the standard deviation.

Several pre-processing steps, including variable imputation, outlier elimination, and removal of highly correlated variables were undertaken (see Appendix). After completing these procedures, 106 variables for each patient remained to be used by the hospitalization model. For the ICU and ventilation prediction models, we added laboratory results and radiologic findings. We removed variables with more than 90% missing values out of the roughly 2500 patients retained for these models; the remaining missing values were imputed as described above. These pre-processing steps retained 130 variables for the ICU and ventilation models.

### Classification methods

We employed nonlinear ensemble methods including Random forests (RF) (Breiman, 2001) and XGBoost (Chen and Guestrin, 2016). We also employed ‘custom’ linear methods which yield interpretable models; specifically, support vector machines (SVM) (Cortes and Vapnik, 1995) and Logistic Regression (LR). In both cases, the variants we computed were robust to noise and the presence of outliers (Chen and Paschalidis, 2018), using proper regularization. LR, in addition to a prediction, provides the likelihood associated with the predicted outcome, which can be used as a confidence measure in decision making. Further details on these methods are in the Appendix.

For each outcome, we used the statistical feature selection and recursive feature elimination procedures described in the Appendix to develop an LR parsimonious model. The LR coefficients are comparable since the variables are standardized. Hence, a larger absolute coefficient indicates that the corresponding variable is a more significant predictor. Positive (negative) coefficients imply positive (negative) correlation with the outcome. We also developed a version of this model by converting all continuous variables into binary variables, using medically motivated thresholds (see Appendix). We report the coefficients of the ‘binarized’ model and the implied odds ratio (OR), representing how the odds of the outcome are scaled by having a specific variable being abnormal vs. normal, while controlling for all other variables in the model.

### Outcomes and performance metrics

Model performance metrics included the Area Under the Curve (AUC) of the Receiver Operating Characteristic (ROC) and the Weighted-F1 score. The ROC plots the true positive rate (a.k.a. recall or sensitivity) against the false positive rate (equal to one minus the specificity). We optimized algorithm parameters to maximize AUC.

The F1 score is the harmonic mean of precision and recall. Precision (or positive predictive value) is defined as the ratio of true positives over true and false positives. The Weighted-F1 score is computed by weighting the F1-score of each class by the number of patients in that class.

### Model validation

The data were split into a training (80%) and a test set (20%). Algorithm parameters were optimized on the training (derivation) set using fivefold cross-validation. Performance metrics were computed on the test set. This process was repeated five times, each time with a random split into training/testing sets. In columns labeled as *Random* in Tables 1, 2, 3, 4, 5, we report the average (and standard deviation) of the test performance metrics over the five random splits. We also performed a different type of validation. We trained the models on MGH, FH, NWH, and NSM patients, and evaluated performance on BWH patients. These results are reported under the columns *BWH* in the tables.

## Data Availability

Source code for processing patient data is provided together with the submission. Due to HIPAA restrictions and Data Use Agreements we can not make the original patient data publicly available. Interested parties may submit a request to obtain access to de-identified data to the authors. The authors would request pertinent IRB approval to make available a de-identified version of the data, stripped of any protected health information as specified under HIPAA rules. The IRB of the hospital system approved the study under Protocol #2020P001112 and the Boston University IRB found the study as being Not Human Subject Research under Protocol #5570X (the BU team worked with a de-identified limited dataset).

## Additional files

Source code 1. COVID-19 Early Prediction Scripts, Network Optimization and Control Lab, Boston University.

Transparent reporting form

## Appendix 1

1. Representative statistics of patients and variables highly correlated with the outcomes Characteristics of the 2566 patients who tested positive for SARC-CoV2 with key statistics for each cohort (hospitalized vs. not, ICU admitted vs. not, and mechanically ventilated vs. not) are provided in Appendix 1—table 1. For each variable we provide a mean value of the variable (or percentage for categorical variables) in each cohort and its complement and a p-value computed using a chi-squared test for categorical variables and a Kolmogorov-Smirnov (KS) test for continuous variables. A low p-value supports rejection of the null hypothesis, implying that the corresponding variable is statistically different in a cohort compared to its complement (e.g., hospitalized vs. not). Appendix 1—table 2 reports how the entire patient cohort is distributed across the five different hospitals according to the various outcome groups.

**Appendix 1—table 1.** Representative patient statistics.

	Admitted (36.2%)			ICU (10.6%)			Intubated (8.5%)		
	Yes	No	p-value	Yes	No	p-value	Yes	No	p-value
Age	62.3	46.0	<0.001	63.3	50.6	<0.001	63.3	50.9	<0.001
Gender (male)	55.3%	40.1%	<0.001	63.0%	43.5%	<0.001	63.6%	43.9%	<0.001
Asian	3.7%	4.0%	0.97	3.7%	3.9%	1	3.7%	3.9%	1
Black/African American	15.7%	17.8%	0.61	14.7%	17.3%	0.75	14.3%	17.3%	0.74
Hispanic/Latino	4.9%	5.9%	0.81	6.6%	5.4%	0.88	6.9%	5.4%	0.83
White	45.4%	43.9%	0.91	39.6%	45.0%	0.40	39.6%	44.9%	0.53
Hypertension	61.7%	26.4%	<0.001	62.3%	36.5%	<0.001	61.8%	37.1%	<0.001
Diabetes	34.2%	9.7%	<0.001	40.7%	15.9%	<0.001	42.9%	16.3%	<0.001
Alzheimer	6.7%	0.6%	<0.001	2.6%	2.8%	1	3.2%	2.7%	0.98
Congestive Heart Failure (CHF)	11.3%	0.8%	<0.001	9.5%	4.0%	<0.001	8.8%	4.2%	0.025
Chronic Kidney Disease (CKD)	14.4%	1.7%	<0.001	12.8%	5.5%	<0.001	11.5%	5.8%	0.011
ACE Inhibitors (ACEIs)	17.5%	8.4%	<0.001	20.5%	10.7%	<0.001	19.8%	11.0%	0.002
Acetaminophen Tylenol	39.8%	17.8%	<0.001	31.9%	25.1%	0.12	30.4%	25.4%	0.45
Amiodarone	1.6%	0.1%	<0.001	1.5%	0.5%	0.32	0.9%	0.6%	0.95
Anticoagulants	9.4%	1.7%	<0.001	9.9%	3.8%	<0.001	11.1%	3.8%	<0.001
Anti-depressants	25.4%	16.7%	<0.001	20.5%	19.8%	0.99	22.6%	19.6%	0.77
Angiotensin Receptor Blockers (ARBs)	12.0%	5.2%	<0.001	15.4%	6.8%	<0.001	17.1%	6.8%	<0.001
Aspirin related	32.3%	11.6%	<0.001	33.7%	17.4%	<0.001	33.2%	17.8%	<0.001
Beta-Blockers	28.1%	10.4%	<0.001	25.6%	15.7%	<0.001	25.8%	16.0%	0.003
Calcium Chanel Blockers (CCBs)	2.6%	0.7%	0.001	4.4%	1.0%	<0.001	4.6%	1.1%	<0.001
Coumadin warfarin	3.5%	0.7%	<0.001	1.8%	1.7%	1	1.8%	1.7%	1
Diuretics	16.0%	4.5%	<0.001	13.9%	8.1%	0.015	13.4%	8.3%	0.089
Immuno- suppressants	5.3%	2.6%	0.005	3.7%	3.5%	1	4.1%	3.5%	0.97
Insulin related	14.6%	3.5%	<0.001	19.0%	6.2%	<0.001	21.2%	6.3%	<0.001
Metformin related	19.5%	8.6%	<0.001	23.8%	11.2%	<0.001	24.9%	11.4%	<0.001
Nonsteroidal anti-inflammatory drugs (NSAIDs)	21.9%	21.0%	0.95	19.0%	21.6%	0.82	18.0%	21.6%	0.66
Proton Pump Inhibitors (PPIs)	26.6%	15.0%	<0.001	24.5%	18.5%	0.13	25.8%	18.6%	0.081
Statins	45.1%	17.3%	<0.001	47.6%	24.9%	<0.001	45.6%	25.7%	<0.001
Steroids	30.5%	23.0%	<0.001	30.8%	25.2%	0.26	30.4%	25.3%	0.44
Cough	65.6%	29.6%	<0.001	68.1%	39.6%	<0.001	69.1%	40.2%	<0.001
Dyspnea	16.6%	2.2%	<0.001	21.6%	5.7%	<0.001	23.5%	5.9%	<0.001
Chest pain	21.1%	5.6%	<0.001	22.0%	9.9%	<0.001	24.4%	10.0%	<0.001
Fever	57.4%	23.7%	<0.001	61.2%	32.9%	<0.001	63.6%	33.4%	<0.001
SpO2	95.2	97.4	<0.001	93.4	96.7	<0.001	93.3	96.7	<0.001
Diastolic BP	72.5	78.1	<0.001	72.0	75.6	<0.001	70.9	75.6	<0.001
Pulse	90.6	88.3	<0.001	93.3	88.8	0.003	94.1	88.9	0.01
Respiratory Rate (RR)	23.1	20.3	<0.001	25.6	21.2	<0.001	25.9	21.3	<0.001
Temperature (oC)	37.2	37.0	<0.001	37.3	37.1	0.001	37.3	37.1	0.001
Anion Gap	15.8			17.0	15.1	<0.001	17.1	15.1	<0.001
Sodium	137.0			136.3	137.4	<0.001	136.2	137.3	<0.001
Calcium	9.0			8.8	9.0	<0.001	8.8	9.0	<0.001
Lactic acid	1.8			2.1	1.6	<0.001	2.1	1.6	<0.001
Glomerular filtration rate (GFR)	67.0			64.8	72.3	<0.001	64.7	71.9	<0.001
Chloride	98.1			97.2	98.8	<0.001	97.1	98.8	<0.001
Glucose	149.6			171.5	135.8	<0.001	173.9	137.2	<0.001
Lactate Dehydrogenase (LDH)	377.2			524.6	303.9	<0.001	551.8	310.6	<0.001
Albumin	3.8			3.6	3.9	<0.001	3.6	3.9	<0.001
D-Dimer	1373.5			1525.0	1223.7	<0.001	1614.5	1214.0	<0.001
C-reactive Protein (CRP)	89.6			133.1	65.5	<0.001	140.1	68.1	<0.001
Blood Urea Nitrogen (BUN)	21.4			24.3	18.5	<0.001	23.8	18.9	<0.001
Creatine Kinase (CK)	385.2			563.4	282.7	<0.001	620.3	285.1	<0.001
Ferritin	854.2			1349.5	601.6	<0.001	1477.1	621.8	<0.001
Mean Platelet Volume (MPV)	10.5			10.6	10.5	<0.001	10.6	10.5	<0.001
Atelectasis	19.0%	4.6%	<0.001	15.8%	9.2%	0.008	16.6%	9.2%	0.007
Consolidation	5.9%	0.6%	<0.001	10.3%	1.6%	<0.001	11.1%	1.7%	<0.001
Nodule	4.9%	0.6%	<0.001	4.4%	1.9%	0.072	3.7%	2.0%	0.47
Opacity	64.8%	13.7%	<0.001	78.4%	26.7%	<0.001	80.6%	27.8%	<0.001
Pleural Effusion	8.8%	1.1%	<0.001	11.7%	3.0%	<0.001	13.8%	3.0%	<0.001

**Appendix 1—table 2.** Distribution of patients in different hospitals and outcome groups.

Hospital	Positive	Admitted	ICU	Intubated
Brigham and Women's Hospital (BWH)	648	171	67	56
Newton-Wellesley Hospital (NWH)	434	145	33	18
Massachusetts General Hospital (MGH)	1195	475	144	121
North Shore Medical Center (NSM)	97	63	16	12
Faulkner Hospital (FH)	192	76	13	10
Total	2566	930	273	217 2. Natural Language Processing (NLP) of clinical notes The de-identified data consisted of demographics, lab results, history and physical examination (H and P) notes, progress notes, radiology reports, and discharge notes. We extracted all variables needed for each patient and built a profile using NLP tools. There were mainly two difficulties. First, many important features such as vitals and medical history (prior conditions, medications) were not in a table format and were extracted from the report text using different regular expression templates, post-processing the results to eliminate errors due to non-uniformity in the reports (e.g., a line break may separate a date from the field indicating the type). Second, the negations in the text should be recognized. Simply recognizing a medical term such as ‘cough’ or ‘fever’ is not sufficient since the report may include ‘Patient denies fever or cough’. We applied multiple NLP schemes to overcome these difficulties. Regular expression matching is the basic strategy we used to extract features such as body temperature values (with or without decimal followed by ‘?C/?F’) and blood pressure values (‘xx(x)/xx(x)’ even if they are mixed up with a date ‘mm/dd/yyyy’ having similar symbols). Extracting pulse and respiratory rates is challenging since it is easy to mismatch the corresponding values; thus, we also matched the indicators ‘RR:’ (respiratory rate) or ‘P’ (pulse rate) in the vicinity of the number. To extract symptoms in H and P notes and findings in radiology reports, we used two NLP models: a Named Entity Recognition (NER) model, and a Natural Language Inference (NLI) model (Zhu et al., 2018). The first model aims at finding all the symptoms/disease named entities in the report. The key motivation of NER is that it is hard to list all possible disease names and search for them in each sentence; instead, NER models use the context to infer the possible targets, thus, even abbreviations like ‘N/V’ will be recognized. We used the spaCy NER model (Kiperwasser and Goldberg, 2016) trained on the BC5CDR corpus. The NLI model is used to detect negations, by checking if a sentence as a premise supports the hypothesis that the patient truly has the disease/symptoms in it. We applied a fine-tuned RoBERTa model (Liu et al., 2019) to perform NLI. For medication extraction, we used the Unified Medical Language System (UMLS) (UMLS, 2019), which comprehensively contains medical terms and their relationships. We added a medication to the patient’s prior to admission medication list only If the medication or brand name is found in the UMLS ‘Pharmacologic Substance’ or ‘Clinical Drug’ category. Symptoms, medical history, and prior medications from H and P notes are often described using different terminology or acronyms that imply the same condition or medication (e.g., dyspnea and SOB). We manually mapped these non-unique descriptors to distinct categories. An appropriate classification was also used for comorbidities, prior medications, radiological findings, and laboratories. The entire list of variables extracted and used in the analysis is provided in Appendix 1—table 3.

**Appendix 1—table 3.** List of 164 features used for hospitalization, ICU, and ventilation models.

Category	Features
Demographics	Marital status, Gender, Race, Age, Language, Tobacco, Alcohol, Height, Weight, BMI
Vitals	Systolic BP, Diastolic BP, Temperature, Pulse, Respiratory Rate, SpO2 percentage
Symptoms	Fever, Cough, Dyspnea, Fatigue, Diarrhea, Nausea, Vomiting, Abdominal pain, Loss of smell, Loss of taste, Chest pain, Headache, Sore throat, Hemoptysis, Myalgia
Pre-existing medications	Steroids, ACEIs, ARBs, NSAIDs, Anti-depressants, CCBs, Diuretics, Digoxin, Statins, Beta-Blockers, Acetaminophen Tylenol, Immunosuppressants, Anticoagulants, Aspirin related, Coumadin warfarin, Amiodarone, Insulin related, Metformin related, PPIs
Comorbidities	Hypertension, COPD, Diabetes, CKD, CAD, MI, Asthma, Osteoarthritis arthritis, SLE, HLD, Arrhythmia, Thyroid disease, Stroke, Migraine, Epilepsy, Alzheimer, Parkinson, Nephrolithiasis, Cushing, Adrenal Insufficiency, Diverticulosis, GERD, IBS, IBD, Cholelithiasis, Inguinal hernia, Hepatitis, Cirrhosis, Valvular disease, CHF, PAD, Osteoporosis, Cancer, TB, Cardiomyopathy, AAA, DVT, vWD, Anemia, Transplantation, HIV, Depression, Anxiety
Radiology	Opacity, Atelectasis, Consolidation, Pleural Effusion, Pneumothorax, Nodule
Labs	RDW, PLT, MCH, HGB, MCHC, HCT, MCV, RBC, WBC, MPV, NRBC (%), GFR (estimated), Creatinine, Potassium, Chloride, Sodium, Anion Gap, BUN, Glucose, Calcium, Carbon Dioxide, Absolute Neutrophil count, Absolute Lymphocyte count, Absolute Monocyte count, Absolute Eosinophil count, Absolute Basophil count, Immature Granulocytes, ALT, Total Protein, Albumin, Globulin, AST, Bilirubin (Total), Alkaline phosphatase, NRBC Auto (#), LDH, Ferritin, CK, Magnesium, CRP, PT, D-Dimer, Lactic acid, Phosphorus, PTT, PCO2 (Venous), pH (Venous), Fibrinogen, Lipase, Bands (manual), PO2 (Venous), Base Deficit (Venous), Iron, Bilirubin (Direct), Myelocytes, HCO3 (unspecified), TIBC, Base Deficit (Arterial), PCO2 (Arterial), Metamyelocytes, Plasma cells (%), PO2 (Arterial), Ionized Calcium, pH (Arterial), Osmolality

To evaluate the accuracy of the NLP models on our data, we randomly selected 35 hr and P notes and manually checked the model, evaluating the precision, recall, and F1-score for the extracted terms. For the NER+NLI deep learning model, we compared all the symptoms extracted by the models against the manually extracted ground truth. For the general regular expression matching models, we checked the extraction of vitals as a representative task, particularly since vitals have the most complicated format in the original notes. Appendix 1—table 4 provides the results of this manual evaluation.

**Appendix 1—table 4.** Performance of the NLP models.

	Precision (%)	Recall (%)	F1-score (%)
NER+NLI model	93.60	87.97	90.70
Regular expression matching	99.01	96.15	97.56

**Appendix 1—table 5.** Abnormal ranges for laboratory tests and vitals.

Variable	Abnormal range
Albumin	<3.3
Chloride	<95
Lactic acid	≥2
LDH	≥250
CRP (mg/L)	≥10
Calcium	≤8.5
Anion gap	≥12
Glucose	≥110
Total protein	≤6.5 or ≥8.3
D-Dimer (ng/mL)	≥500
GFR	≤60
Sodium	<135
Globulin	≤2 or ≥4
SpO2	≤94
Systolic blood pressure	≤100
Pulse	≥100
Respiratory rate	≥20
Age	≥65
Diastolic blood pressure	≤60
BMI	≥30
Temperature	≥37.5 °C or ≥98.7 °F

For both types of models, the F1-score exceeds 90%. Most of the symptoms missing are due to non-obvious abbreviations. Regular expression matching has better performance since potential errors may only come from very rare formats we did not consider. 3. Classification methods A random forest (RF) (Breiman, 2001) is an ensemble algorithm that achieves high accuracy and generalization performance by combining multiple weak decision tree classifiers. For training, RF uses bootstrap aggregating (bagging) technique to randomly select a training sample set for each decision tree classifier. It trains multiple decision trees in parallel during the training phase, where each tree is trained using a random sample set from the original training set. In the test phase, RF uses the trained decision tree classifiers to classify a test sample, and then combines all the classifiers by majority voting. XGBoost (Chen and Guestrin, 2016) generates a series of decision trees in sequential order; each decision tree is fitted to the residual between the prediction of the previous decision tree and the target value, and this is repeated until a predetermined number of trees or a convergence criterion is reached. All decision trees computed are combined with proper weights to produce a final decision. XGBoost uses shrinkage and column subsampling to prevent overfitting and achieves fast training using a number or parallelization approaches. Both of these nonlinear models are expensive to train compared to the linear models we discuss next. Essentially, each one of them trains an ensemble of many decision trees (could be as many as 500 or more) and a decision is made by combining information from all of these trees. Among the linear classifiers, we used the support vector machine (SVM) (Cortes and Vapnik, 1995), which computes an optimal hyperplane separating the two classes. To render the method robust to noise and the presence of outliers (Chen and Paschalidis, 2018) we used (ℓ_1_- or ℓ_2_-norm) regularized versions of SVM. We also used Logistic regression (LR) -- a common classification method that uses a linear regression model to approximate the logarithmic odds (logit) of the true classification label. LR, in addition to a prediction, also provides the likelihood of the predicted outcome, which can be used as a confidence measure in decision making. Similar to SVM, we used (ℓ_1_- or ℓ_2_-norm) regularized logistic regression to find the optimal subset of features from the initial feature space. In particular, based on the LR model, the predicted probability of the outcome, denoted by $\hat{y}$, is estimated by the formula:

$$\hat{y}=\frac{1}{1+\;exp\left\{-b_{0}-\sum_{i=1}^{n}b_{i}x_{i}\right\}},$$

where exp{⋅} denotes the exponential function, $b_{0}$ is the intercept, $\left(x_{1},\ldots ,x_{n}\right)$ the variables used by the model, and $\left(b_{1},\ldots ,b_{n}\right)$ the corresponding coefficients. Using this formula and the LR coefficients (and intercept) provided in Tables 1, 2, 3, 4, 5, one can obtain an easily computable value for the predicted probability of the corresponding outcome. Comparing that value to a threshold (in the interval [0,1]) yields a prediction. The threshold can be set depending on the desired trade-off between sensitivity and specificity, which is typically specified by the user. 4. Pre-processing, statistical feature selection and recursive feature elimination We extracted patients’ laboratory test results at the date of hospital admission (reference date). Since some lab tests may be received several hours after the reference time, we extracted the nearest set of lab results to the reference time. Some tests have multiple Logical Observation Identifiers Names and Codes (LOINC), referring to the same quantity, and were merged. White blood cells (WBC) types (basophils, eosinophils, lymphocytes, monocytes, and neutrophils) were reported both as an absolute count and percentage (of WBC). We eliminated the percentages and maintained the absolute counts. We also removed all laboratory test results that did not contain enough information for a significant percentage of the patients (less than 10%). This retained 65 laboratory variables. Missing variables were imputed using the mode or, for some key lab variables, by regressing on the non-missing variables of the patient. To mitigate the effect of outliers, each variable with values higher than the 99th percentile or lower than the 1st percentile, was replaced with the 99th or 1st percentile, respectively. Finally, and to avoid collinearity, of the variables that were highly correlated (absolute correlation coefficient higher than 0.8) we removed one among the two. For each model, we used a variety of statistical feature selection approaches. Specifically, we first calculated a p-value for each variable as described earlier and removed all variables with a p-value exceeding 0.05. Further, we used (ℓ_1_-norm) regularized LR and performed recursive feature elimination as follows. We run LR and obtained the coefficients of the model. We then eliminated the variable with the smallest absolute coefficient and re-run LR to obtain a new model. We kept iterating in this fashion, to select a model that maximizes a metric equal to the mean AUC minus its standard deviation in a validation dataset. 5. Thresholds for the binarized models Thresholds used for generating binarized versions of our parsimonious models are reported in Appendix 1—table 5. In these models, a variable is set to one if the corresponding continuous variable is abnormal and 0 otherwise. 6. Standard pneumonia severity scores For comparison purposes we implemented two commonly used pneumonia severity scores, CURB-65 (Lim et al., 2003) and the Pneumonia Severity Index (PSI) (Fine et al., 1997). CURB-65 uses a mental test assessment, Blood Urea Nitrogen (BUN), respiratory rate, blood pressure, and the indicator of age being 65 or older. PSI uses similar information, a host of laboratory values, and comorbidities. From CURB-65 we did not score for mental status since we did not have such information. From PSI, we did not use mental status and whether the patient was a nursing home resident. Given that laboratory values are used, we computed these scores to predict ICU care and ventilator use. In each case, we computed the corresponding score and then optimized a threshold using cross-validation over the training set in order to make the prediction. We used these thresholds and evaluated performance of each scoring system in the test set. 7. Training/Derivation Model Performance Performance metrics for the various models on the training/derivation cohorts are reported in Appendix 1—tables 6, 7, 8, 9, 10. These are computed for both the random splitting of the data into training and testing sets (in this case, we provide the mean and standard deviation over the five random splits), as well as for the training dataset formed from patients at MGH, FH, NWH, and NSM (these results are under the column named BWH in Appendix 1—tables 6, 7, 8, 9, 10, simply to match the terminology of Tables 1, 2, 3, 4, 5).

**Appendix 1—table 6.** Derivation cohort performance for the hospitalization prediction model. Abbreviations and metrics reported are as in Table 1.

Algorithm	AUC		F1-weighted	
	Random	BWH	Random	BWH
	Models using all 106 features			
LR-L2	88.3% (0.4%)	88.3%	82.9% (0.5%)	82.3%
SVM-L1	88.2% (0.4%)	88.2%	82.8% (0.5%)	82.1%
XGBoost	91.5% (2.1%)	90.9%	85.7% (2.3%)	85.2%
RF	96.0% (0.7%)	95.3%	92.9% (1.2%)	90.8%
	Models using 74 statistically selected features			
LR-L2	87.8% (0.4%)	87.8%	82.4% (0.4%)	81.7%
SVM-L1	87.8% (0.4%)	87.7%	82.5% (0.7%)	81.7%
XGBoost	91.9% (1.8%)	91.9%	86.0% (1.8%)	86.2%
RF	94.9% (0.9%)	96.6%	91.3% (1.3%)	93.2%
	Parsimonious Model using 11 features			
LR-L2	82.6% (0.5%)	82.4%	77.6% (0.1%)	76.9%
SVM-L1	82.5% (0.5%)	82.3%	77.5% (0.3%)	76.9%

**Appendix 1—table 7.** Derivation cohort performance for the ICU prediction model. Abbreviations and metrics reported are as in Table 1.

ICU prediction results (training performance) with 2513 patients				
Algorithm	AUC		F1-weighted	
	Random	BWH	Random	BWH
	Models using all 130 features			
XGBoost	94.5% (3.6%)	96.1%	94.0% (1.7%)	94.1%
SVM-L1	89.7% (0.7%)	91.4%	91.5% (0.4%)	91.9%
LR-L1	91.3% (0.6%)	92.9%	91.5% (0.5%)	91.9%
RF	93.4% (3.2%)	97.0%	94.3% (1.6%)	95.4%
	Models using 56 statistically selected features			
XGBoost	94.1% (1.5%)	95.1%	93.6% (0.6%)	93.7%
SVM-L1	88.5% (0.7%)	89.7%	91.2% (0.4%)	91.4%
LR-L1	89.3% (0.7%)	90.4%	91.2% (0.2%)	91.4%
RF	91.0% (1.9%)	94.9%	93.0% (1.0%)	94.2%
	Parsimonious Model using 10 features			
LR-L1	86.2% (0.6%)	83.8%	90.4% (0.4%)	89.1%
LR-L1 (binarized model)	84.0% (0.6%)	80.6%	89.4% (0.1%)	88.2%
	Model using PSI or CURB-65 score			
PSI score	74.3% (1.2%)	72.3%	87.5% (0.2%)	87.1%
CURB-65 score	67.9% (1.3%)	65.3%	87.3% (0.2%)	86.8%

**Appendix 1—table 8.** Derivation cohort performance for the restricted ICU prediction model. Abbreviations and metrics reported are as in Table 1.

ICU prediction training performance with 628 patients				
Algorithm	AUC		F1-weighted	
	Random	BWH	Random	BWH
	Models using all 130 features			
XGBoost	89.6% (4.8%)	92.5%	85.4% (5.8%)	87.6%
SVM-L1	80.1% (0.6%)	80.8%	79.4% (0.5%)	80.4%
LR-L1	87.1% (0.8%)	88.0%	83.5% (0.5%)	83.6%
RF	95.6% (2.9%)	95.7%	91.0% (3.3%)	90.2%
	Models using 29 statistically selected features			
XGBoost	86.3% (1.0%)	87.4%	81.9% (0.4%)	83.8%
SVM-L1	80.5% (0.9%)	80.4%	79.1% (0.5%)	80.4%
LR-L1	80.9% (1.0%)	81.6%	79.0% (0.3%)	80.3%
RF	89.8% (2.6%)	92.8%	85.0% (1.9%)	88.2%
	Parsimonious Model using 8 features			
LR-L1	80.4% (0.9%)	81.4%	79.7% (0.5%)	80.0%
LR-L1 (binarized model)	75.4% (1.1%)	77.2%	75.2% (0.7%)	77.5%
	Model using PSI or CURB-65 score			
PSI score	60.5% (1.7%)	59.0%	68.6% (0.5%)	68.7%
CURB-65 score	60.2% (1.2%)	57.2%	67.5% (0.4%)	67.3%

**Appendix 1—table 9.** Derivation cohort performance for the ventilation prediction model. Abbreviations and metrics reported are as in Table 1.

Ventilation prediction training performance with 2525 patients				
Algorithm	AUC		F1-weighted	
	Random	BWH	Random	BWH
	Models using all 130 features			
XGBoost	97.2% (1.5%)	95.2%	95.8% (1.0%)	94.5%
SVM-L1	92.3% (0.7%)	92.8%	93.1% (0.1%)	93.4%
LR-L1	93.8% (0.6%)	94.3%	93.3% (0.2%)	93.2%
RF	95.1% (0.8%)	94.7%	95.4% (0.5%)	94.3%
	Models using 55 statistically selected features			
XGBoost	96.9% (1.4%)	98.3%	95.6% (0.9%)	96.6%
SVM-L1	90.8% (0.7%)	91.3%	92.7% (0.2%)	93.0%
LR-L1	91.4% (0.7%)	92.0%	92.6% (0.3%)	92.8%
RF	94.8% (0.7%)	94.1%	95.5% (0.3%)	94.8%
	Parsimonious Model using 8 features			
LR-L1	86.9% (0.5%)	88.1%	91.6% (0.2%)	91.9%
LR-L1 (binarized model)	84.4% (0.7%)	86.7%	91.1% (0.2%)	91.2%
	Model using PSI or CURB-65 score			
PSI score	74.0% (1.0%)	71.4%	89.9% (0.1%)	89.6%
CURB-65 score	67.6% (0.8%)	64.7%	89.7% (0.0%)	89.6%

**Appendix 1—table 10.** Derivation cohort performance for the restricted ventilation prediction model. Abbreviations and metrics reported are as in Table 1.

Ventilation prediction training performance with 635 patients				
Algorithm	AUC		F1-weighted	
	Random	BWH	Random	BWH
	Models using all 130 features			
XGBoost	91.8% (2.2%)	98.6%	87.4% (2.0%)	95.3%
SVM-L1	81.2% (0.7%)	83.2%	82.4% (1.1%)	83.9%
LR-L1	89.7% (0.6%)	89.6%	86.9% (1.0%)	85.8%
RF	93.5% (4.2%)	93.7%	89.5% (3.8%)	89.7%
	Models using 29 statistically selected features			
XGBoost	89.9% (2.3%)	89.9%	86.1% (1.6%)	86.0%
SVM-L1	81.5% (1.6%)	84.4%	82.2% (1.2%)	83.7%
LR-L1	82.6% (0.7%)	84.0%	83.0% (0.9%)	83.6%
RF	92.3% (4.8%)	94.3%	88.8% (3.7%)	89.3%
	Parsimonious Model using 5 features			
LR-L1	80.3% (1.0%)	79.0%	82.1% (0.7%)	81.7%
LR-L1 (binarized model)	73.1% (1.4%)	66.5%	78.3% (0.9%)	73.5%
	Model using PSI or CURB-65 score			
PSI score	58.8% (1.0%)	57.2%	73.9% (0.3%)	74.2%
CURB-65 score	58.5% (1.7%)	55.8%	73.2% (0.1%)	73.7% 8. Performance of the restricted ICU and ventilation models with sufficient distance to the event Appendix 1—table 11 lists the performance of the restricted ICU and mechanical ventilation parsimonious LR-L1 models provided in Tables 3 and 5 when applied to a test set consisting of the BWH patients and 11 additional patients whose data were collected right after the original dataset was compiled. In these results, we excluded patients whose predicted outcome (ICU or intubation) occurs less than x hours from the time the admission lab results were made available, where x takes values in the set {6 hr, 12 hr, 18 hr, 24 hr, 48 hr}. Thus, the corresponding test set includes only patients with sufficient time difference from the data used to make the prediction, assessing how far into the future the predictive model could reach. We added the additional 11 patients to make sure we have a sufficient number of test patients to perform this study. As the results suggest, ICU admission estimation is fairly accurate and robust, whereas intubation prediction had moderate predictive power.

**Appendix 1—table 11.** AUC and weighted F1-score on an extended BWH test set, where patients with lab-to outcome time smaller than or equal to certain gaps are excluded.

Time gap	6hr	12 hr	18 hr	24 hr	48 hr
Restricted ICU model - AUC	86.05%	84.73%	86.85%	86.14%	84.62%
Restricted ICU model - weighted-F1	83.10%	82.17%	86.47%	86.09%	86.28%
Restricted intubation model - AUC	68.00%	64.44%	63.85%	63.85%	64.34%
Restricted intubation model - weighted-F1	65.75%	66.59%	69.81%	69.81%	72.33%
