# Supplementary material for: Early prediction of level-of-care requirements in patients with COVID-19
Source: eLife. 2020 Oct 12;9:e60519. doi: 10.7554/eLife.60519 (PMC7595731; doi:10.7554/eLife.60519)
Supplement: Transparent reporting form [file elife-60519-transrepform.docx]

***eLife’s* transparent reporting form**

We encourage authors to provide detailed information *within their submission* to facilitate the interpretation and replication of experiments. Authors can upload supporting documentation to indicate the use of appropriate reporting guidelines for health-related research (see [EQUATOR Network](about:blank)), life science research (see the BioSharing Information Resource), or the [ARRIVE guidelines](about:blank) for reporting work involving animal research. Where applicable, authors should refer to any relevant reporting standards documents in this form.

If you have any questions, please consult our Journal Policies and/or contact us: [editorial@elifesciences.org](about:blank).

**Sample-size estimation**

- You should state whether an appropriate sample size was computed when the study was being designed
- You should state the statistical method of sample size computation and any required assumptions
- If no explicit power analysis was used, you should describe how you decided what sample (replicate) size (number) to use

Please outline where this information can be found within the submission (e.g., sections or figure legends), or explain why this information doesn’t apply to your submission:

The sample size is mentioned in the abstract and in Sec. 2.1, with more details on how samples were obtained. The number of samples is n=2,566 and it is what was available at the time data were extracted (from the beginning of the COVID-19 outbreak in Massachusetts until Apr. 13 2020).

**Replicates**

- You should report how often each experiment was performed
- You should include a definition of biological versus technical replication
- The data obtained should be provided and sufficient information should be provided to indicate the number of independent biological and/or technical replicates
- If you encountered any outliers, you should describe how these were handled
- Criteria for exclusion/inclusion of data should be clearly stated
- High-throughput sequence data should be uploaded before submission, with a private link for reviewers provided (these are available from both GEO and ArrayExpress)

Please outline where this information can be found within the submission (e.g., sections or figure legends), or explain why this information doesn’t apply to your submission:

- Validation procedures are described in Sec. 2.6, where it is stated that the random validation was performed 5 times and statistics (mean and std) over these runs were computed.
- Data pre-processing procedures are described in Sec. 2.2 and which patients included in each model in Sec. 2.3.
- Outlier elimination in described in Sec. 4 of the Appendix.

**Statistical reporting**

- Statistical analysis methods should be described and justified
- Raw data should be presented in figures whenever informative to do so (typically when N per group is less than 10)
- For each experiment, you should identify the statistical tests used, exact values of N, definitions of center, methods of multiple test correction, and dispersion and precision measures (e.g., mean, median, SD, SEM, confidence intervals; and, for the major substantive results, a measure of effect size (e.g., Pearson's r, Cohen's d)
- Report exact p-values wherever possible alongside the summary statistics and 95% confidence intervals. These should be reported for all key questions and not only when the p-value is less than 0.05.

Please outline where this information can be found within the submission (e.g., sections or figure legends), or explain why this information doesn’t apply to your submission:

- Statistical methods used for variable selection and classification models are described in Sec. 2, with more details provided in the Appendix (Secs. 1, 3, and 4).
- A description on how p-values were computed is in the Appendix (Sec. 1).
- Standard deviations for performance statistics, odds ratios, and other quantities are reported in Tables 1-5 and in the text.

(For large datasets, or papers with a very large number of statistical tests, you may upload a single table file with tests, Ns, etc., with reference to sections in the manuscript.)

**Group allocation**

- Indicate how samples were allocated into experimental groups (in the case of clinical studies, please specify allocation to treatment method); if randomization was used, please also state if restricted randomization was applied
- Indicate if masking was used during group allocation, data collection and/or data analysis

Please outline where this information can be found within the submission (e.g., sections or figure legends), or explain why this information doesn’t apply to your submission:

- Validation procedures outlined in Sec. 2.6 describe how samples were allocated into training and test cohorts.

**Additional data files (“source data”)**

- We encourage you to upload relevant additional data files, such as numerical data that are represented as a graph in a figure, or as a summary table
- Where provided, these should be in the most useful format, and they can be uploaded as “Source data” files linked to a main figure or table
- Include model definition files including the full list of parameters used
- Include code used for data analysis (e.g., R, MatLab)
- Avoid stating that data files are “available upon request”

Please indicate the figures or tables for which source data files have been provided:

- We cannot provide the data we used because they are covered by HIPAA rules and we have signed very restrictive DUAs with the hospitals that provided the data.
- We cannot even provide a de-identified version of the data because they contain dates (it is a HIPAA limited dataset) and dates are important in our study as they determine when an individual tested positive, was hospitalized, admitted into ICU, intubated, etc. Dates are considered a HIPAA identifier because they could potentially be matched with other records to identify a patient.
- We are submitting however our (python) source code for processing the data and obtaining our results.
- Informed consent was not obtained because the (i) study posed no more than minimal risk to subjects since it examined data retrospectively; and (ii) the research could not have been practically carried out otherwise since it concerned a large number of subjects, many never seen at the hospital (only tested at a testing facility), many after their hospitalization ended, and some potentially deceased.
- The IRB of the hospital system approved the study under Protocol #2020P001112 and the Boston University IRB found the study as being Not Human Subject Research under Protocol #5570X (the BU team worked with a de-identified limited dataset).
