# Supplementary material for: Early prediction of level-of-care requirements in patients with COVID-19
Source: eLife. 2020 Oct 12;9:e60519. doi: 10.7554/eLife.60519 (PMC7595731; doi:10.7554/eLife.60519)
Supplement: Peer review history

# Decision letter

**DOI:** 10.7554/eLife.60519.sa1

**Affiliations:** Attikon University HospitalGreece; Attikon University HospitalGreece

In the interests of transparency, eLife publishes the most substantive revision requests and the accompanying author responses.

**Acceptance summary:**

The paper provides meaningful information on the risk factors for ICU admission and mechanical ventilation. The authors used data from the largest healthcare system in Massachusetts to develop models to predict hospitalization, ICU admission, and need for mechanical ventilation in patients presenting with COVID-19.

**Decision letter after peer review:**

Thank you for submitting your article "Early prediction of level-of-care requirements in patients with COVID-19" for consideration by *eLife*. Your article has been reviewed by two peer reviewers, including Evangelos J Giamarellos-Bourboulis as the Reviewing Editor and Reviewer #1, and the evaluation has been overseen by Jos van der Meer as the Senior Editor.

The reviewers have discussed the reviews with one another and the Reviewing Editor has drafted this decision to help you prepare a revised submission.

As the editors have judged that your manuscript is of interest, but as described below that additional experiments are required before it is published, we would like to draw your attention to changes in our revision policy that we have made in response to COVID-19 (https://elifesciences.org/articles/57162). First, because many researchers have temporarily lost access to the labs, we will give authors as much time as they need to submit revised manuscripts. We are also offering, if you choose, to post the manuscript to bioRxiv (if it is not already there) along with this decision letter and a formal designation that the manuscript is "in revision at *eLife*". Please let us know if you would like to pursue this option. (If your work is more suitable for medRxiv, you will need to post the preprint yourself, as the mechanisms for us to do so are still in development.)

This paper provides potentially meaningful information about the risk factors for ICU admission and mechanical ventilation. The authors used data from the largest healthcare system in Massachusetts to develop models to predict hospitalization, ICU admission, and need for mechanical ventilation in patients presenting with COVID-19.

Essential revisions:

1) The authors use three main outcomes for their prediction: hospitalization, ICU admission and mechanical ventilation (MV). From these outcomes, the authors need to explain how their model is superior over the traditional medical approach of an ICU physician. We doubt that one such model is superior to clinical judgment on the need of hospital admission or ICU admission. The authors need to provide evidence that patients who were missed from clinical judgment cannot be missed from their models and vice versa.

2) The equation of the models is not clear, although the regression factors are provided.

3) What does "a comorbidity of adrenal insufficiency" mean?

4) The results of the derivation and confirmation cohorts should be provided side by side.

5) How earlier does the model predict the need for MV?

6) Subsection “Data description” and Supplementary Table 2: The Pearson correlation coefficient is not a meaningful way to measure the correlation of binary data. A chi-square test or a logit model would be more appropriate.

7) Subsection “Pre-processing and variable selection”. The authors state that they have extracted 164 features, and after preprocessing they retained 106 variables for the hospitalization model and 130 variables for ICU and ventilation. More information about the variables used should be given. Were similar variables grouped together? For example, pyrexia, fever and febrile are used interchangeably in patient notes. The extracted variables should be provided in a supplementary table, and some statistics should be given for these variables (number of missing values, mean, sd, etc) so that the reader can understand and assess the significance of the variables used.

8) Appendix subsection “Representative statistics of patients and variables highly correlated with the outcomes” and Appendix 1—table 1. A t-test (difference between two means) should not be used for binary variables. A chi-squared test or fisher's exact test could be used instead.

9) The authors used natural language processing to extract vitals, medical history, medications, and symptoms. However, they do not mention if they performed (and how) a validation of the NLP model and if they evaluated the correctness of the extracted variables. Was a subset of the extraction manually checked for correctness? Was the extracted medical history compared to coded ICD-10 codes? More information should be given, and the authors need to at least discuss the limitations of the method.

10) The authors state that they "calculated a p-value for each variable as described earlier". In the Appendix subsection “Representative statistics of patients and variables highly correlated with the outcomes” they state that they computed a p-value using a two-sited t-test for binary variables, which is inappropriate (see comment 3). If this is what they did (which is also suggested by the p-values in Table 1), the feature selection process needs to be repeated using the appropriate statistical tests, and the models re-run

11) The authors should discuss the sparsity of the variables and the efficiency of Random forests on sparse data compared to XGBoost.

12) The authors should mention when and why they used each model. Why wasn't XGBoost used in the 'big' ICU prediction model and why RF wasn't used in the restricted ICU model using all 130 features? Ideally, all model architectures should be compared for the reader to be able to understand when to use each model, and for consistency.

13) Abstract and Discussion. "Complex disease" should be rephrased or defined well. We understand that these patients were just hospitalized, and authors give no information on the severity of their disease (for example if these patients required oxygen supplementation, their SpO2 on presentation, etc.)

14) The authors state that they employed "custom" linear methods. Did the authors use a custom loss function in addition to l1 regularization?

# Reply: Author response

**DOI:** 10.7554/eLife.60519.sa2

Essential revisions:

1) The authors use three main outcomes for their prediction: hospitalization, ICU admission and mechanical ventilation (MV). From these outcomes, the authors need to explain how their model is superior over the traditional medical approach of an ICU physician. We doubt that one such model is superior to clinical judgment on the need of hospital admission or ICU admission. The authors need to provide evidence that patients who were missed from clinical judgment cannot be missed from their models and vice versa.

We agree this is a concern. The prediction models are not meant to replace clinicians’ judgment for determining level of care. Instead, they are designed to assist clinicians in identifying patients at risk of future decompensation. In an effort to evaluate the utility of the models to that end, we performed several additional tasks.

1) We tested how accurate are the restricted ventilation and ICU models on a test set of patients with a long interval between the admission lab results used by the model and the corresponding outcome. We considered time lags anywhere from 6h to 48h. In this way we aimed at confirming that the model predicted outcomes long before they became clinically obvious and, therefore, could assist clinicians in designing care early on. Appendix subsection “Performance of the restricted ICU and ventilation models with sufficient distance to the event” and Appendix 1—table 11 describe the results we obtained. We added relevant comments in the Discussion: “These results demonstrate that the predictive models can indeed make predictions well into the future, when physicians would be less certain about the course of the disease and when there is potentially enough time to intervene and improve outcomes.”

2) We also performed a manual review of the models’ predictions. As we report in a passage we added to the Discussion, we identified a number of patients who, despite presenting with mild disease, required ICU admission several days later. Hypothetically, these were patients for whom early clinical assessment might not predict the need for profound deterioration and need of critical care at a later stage. However, our models predicted ICU admission for these patients accurately. We listed a couple of such cases as examples (Discussion, ninth paragraph).

An additional point refers to the fact that different hospitals have varying degrees of expertise and success in handling such a complex disease. COVID-19 is a global disease, which implies that hospitals all over the world, including under-developed countries and rural areas in developed countries, have to care for such patients. While the models may not surpass the experience of top experts in the best medical centers, they could be useful to hospitals with less expertise and resources.

2) The equation of the models is not clear, although the regression factors are provided.

In the Appendix we added a generic formula (subsection “Classification methods”) for Logistic Regression models, which are the parsimonious models we propose in each case and whose coefficients are provided in Tables 1—5. One can then easily obtain the probability of a specific outcome (e.g., ICU admission) for a new patient by using the coefficients of the models we provide and the corresponding variables of such patient.

3) What does "a comorbidity of adrenal insufficiency" mean?

A comorbidity of adrenal insufficiency was defined based on a prior diagnosis of adrenal insufficiency in patients’ charts. All forms of adrenal insufficiency (primary, secondary, and tertiary) were considered. Most patients who were diagnosed with adrenal insufficiency in our cohort had secondary (or pituitary-dependent) adrenal insufficiency.

4) The results of the derivation and confirmation cohorts should be provided side by side.

We have added model performance metrics computed on the training sets (see Appendix subsection “Training/Derivation Model Performance” and Appendix 1—tables 6-10). We elected to add this information in the Appendix to avoid complicating Tables 1—5.

5) How earlier does the model predict the need for MV?

We have added a paragraph to the Discussion, in the Appendix subsection “Performance of the restricted ICU and ventilation models with sufficient distance to the event”, and appendix 1—table 11, to provide such statistics. On average, the restricted ICU and MV models make these predictions 38 and 35 hours in advance.

6) Subsection “Data description” and Supplementary Table 2: The Pearson correlation coefficient is not a meaningful way to measure the correlation of binary data. A chi-square test or a logit model would be more appropriate.

Following your suggestions, we removed this table and computed p-values using a chi-squared test for categorical variables. This information is provided in Appendix 1—table 1.

7) Subsection “Pre-processing and variable selection”. The authors state that they have extracted 164 features, and after pre-processing they retained 106 variables for the hospitalization model and 130 variables for ICU and ventilation. More information about the variables used should be given. Were similar variables grouped together? For example, pyrexia, fever and febrile are used interchangeably in patient notes. The extracted variables should be provided in a supplementary table, and some statistics should be given for these variables (number of missing values, mean, sd, etc) so that the reader can understand and assess the significance of the variables used.

We have made targeted changes in the main paper and the Appendix to explain how variables were extracted and eliminated.

– In the Appendix, subsection “Natural Language Processing (NLP) of clinical notes”, we describe how symptoms, comorbidities, and prior medications were classified into distinct classes in order to avoid using multiple variables for terms that refer to the same condition.

– In the subsection “Hospitalization” we added details on how from the full list of 106 variables for hospitalization we derived a set of 74 variables (using statistical variable selection) and then a much smaller set of 11 variables (using recursive variable elimination). The ICU and ventilation models use all variables of the hospitalization model plus labs. Appendix subsection “Natural Language Processing (NLP) of clinical notes” details the NLP extraction procedures.

– Finally, Appendix 1—table 3 contains the entire list of variables we extracted and used in our analysis.

8) Appendix subsection “Representative statistics of patients and variables highly correlated with the outcomes” and Appendix 1—table 1. A t-test (difference between two means) should not be used for binary variables. A chi-squared test or fisher's exact test could be used instead.

We have addressed this issue and updated all results. We are now using a chi-squared test for categorical variables and a KS test for continuous variables. Performance metrics have not been affected significantly and variables for the parsimonious models are similar.

9) The authors used natural language processing to extract vitals, medical history, medications, and symptoms. However, they do not mention if they performed (and how) a validation of the NLP model and if they evaluated the correctness of the extracted variables. Was a subset of the extraction manually checked for correctness? Was the extracted medical history compared to coded ICD-10 codes? More information should be given, and the authors need to at least discuss the limitations of the method.

In the Appendix subsection “Natural Language Processing (NLP) of clinical notes” and the associated new Appendix 1—table 3 we added results from comparing the NLP results with manual extraction in a subset of the records. Unfortunately, we did not have access to structured EHRs (with database access to diagnoses, prior conditions, drugs, etc.) and ICD9 or ICD10 codes. First, some of the patients may have been seen for the first time by this particular hospital system and an EHR history for them may not have been readily available. Moreover, the ICD-9/10 codes are typically assigned by coders/billers on the administrative side of the hospital and may not necessarily reflect accurately the patient’s condition. A standing criticism of retrospective studies relying on ICD-9/10 is that the characterization of disease may be inadequate. The only data we had access to were tabular records with demographics and labs and text records with H&P notes, progress notes, radiology reports, and discharge notes.

10) The authors state that they "calculated a p-value for each variable as described earlier". In the subsection “Representative statistics of patients and variables highly correlated with the outcomes” they state that they computed a p-value using a two-sited t-test for binary variables, which is inappropriate (see comment 3). If this is what they did (which is also suggested by the p-values in Table 1), the feature selection process needs to be repeated using the appropriate statistical tests, and the models re-run

Addressed in item 8 above.

11) The authors should discuss the sparsity of the variables and the efficiency of Random forests on sparse data compared to XGBoost.

Random forests and XGBoost are similar in terms of computational efficiency and both are substantially more computationally expensive than the linear models we used. We have added a comment to that effect in the Appendix subsection “Classification methods”. We used the word “sparse” not to characterize the training data but the models. Specifically, the goal of the recursive variable elimination procedure we apply (cf. the last paragraph of Appendix subsection “Pre-processing, statistical feature selection and recursive feature elimination”) is to yield a “sparse” model, i.e., a model that utilizes the smallest number of variables without compromising out-of-sample performance. We removed the word “sparse” from the revised paper to avoid any confusion.

12) The authors should mention when and why they used each model. Why wasn't XGBoost used in the 'big' ICU prediction model and why RF wasn't used in the restricted ICU model using all 130 features? Ideally, all model architectures should be compared for the reader to be able to understand when to use each model, and for consistency.

We did use all models in all cases. We simply reported the best nonlinear and the best linear model in each case to decongest the tables. Following your suggestion, we have revised the performance Tables 1—5 and are now reporting for each model the performance of: RF, XGBoost, the best LR model, and the best SVM model.

13) Abstract and Discussion. "Complex disease" should be rephrased or defined well. We understand that these patients were just hospitalized, and authors give no information on the severity of their disease (for example if these patients required oxygen supplementation, their SpO2 on presentation, etc.)

We have clarified in the Discussion what we mean by “complex disease”. This restricted set of patients includes those in need of hospitalization and without missing values in the crucial lab results (specifically LDH and CRP which emerged as important from our models). We have assumed that the availability of all the lab results implies a higher level of complexity for these patients because detailed lab results are typically drawn on patients with more severe symptoms. We modified the term “complex” in the Abstract to “more complex”, as there is not enough space to explain in detail, but we have explained the term sufficiently in the body of the manuscript.

14) The authors state that they employed "custom" linear methods. Did the authors use a custom loss function in addition to l1 regularization?

By “custom” we mean that proper regularization (either ℓ_1_ or ℓ_2_) with cross-validated constant multiplier has been added. The loss function is standard, i.e., hinge loss for SVM and log-likelihood for LR.
